# Herbal medicines modulate gut microbiota in metabolic diseases: a review

**DOI:** 10.3389/fmicb.2026.1833443

**Published:** 2026-06-25

**Authors:** Xin Lan, Yuankui Wei, Yuanyu Zhao, Yong Lai

**Affiliations:** 1 Department of Pathology, Chengdu Integrated TCM and Western Medicine Hospital, Chengdu, Sichuan, China; 2 Institute of Traditional, Chinese Medicine of Sichuan Academy of Chinese Medicine Sciences, Chengdu, Sichuan, China; 3 Institute of Laboratory Animal Sciences, Sichuan Provincial People’s Hospital, University of Electronic Science and Technology of China, Chengdu, China

**Keywords:** bile acids, dysbiosis, insulin resistance, short-chain fatty acid, traditional Chinese medicine

## Abstract

**Background:**

Metabolic diseases—including obesity, type 2 diabetes mellitus (T2DM), and non-alcoholic fatty liver disease (NAFLD)—affect over 1 billion individuals globally and are characterized by insulin resistance, chronic inflammation, and gut microbiota dysbiosis. Herbal medicines offer multi-component therapeutic potential through microbiota modulation, but mechanistic insights remain fragmented.

**Objective:**

This review synthesizes recent advances in herbal medicine-mediated gut microbiota regulation in metabolic diseases and delineates underlying molecular mechanisms.

**Methods:**

A comprehensive literature search was conducted across PubMed and Web of Science. Search strategies employed MeSH terms and free-text keywords encompassing herbal medicines, gut microbiota, and metabolic diseases. Two authors performed study selection and data extraction. Evidence synthesis was structured according to intervention type and metabolic disease category.

**Results:**

Herbal polysaccharides and other compounds consistently increased beneficial bacteria and promoted short-chain fatty acids (SCFAs) production, improving intestinal barrier integrity via ZO-1/Occludin upregulation and attenuating TLR4/NF-κB-mediated inflammation. Herbal formulations exerted synergistic effects by remodeling microbial community structure, correcting SCFA/bile acid imbalances, and activating IRS1/PI3K/AKT insulin signaling. Notably, *Lactobacillus* and *Akkermansia* emerged as recurrent beneficial targets across multiple herbal interventions. However, evidence is predominantly preclinical, and translational validity to humans requires further validation.

**Conclusion:**

Herbal medicines ameliorate metabolic diseases through multi-target gut microbiota modulation, involving SCFA production, bile acid metabolism, and inflammatory pathway attenuation. These mechanistic insights support the development of microbiota-targeted herbal therapeutics, though clinical translation necessitates standardized formulations and rigorous human trials.

## 1 Introduction

A diverse array of conditions under the umbrella of metabolic diseases, including obesity, type 2 diabetes mellitus (T2DM), and non-alcoholic fatty liver disease (NAFLD), are posing significant threats to human health (Wang P. X. et al., 2020; Croci et al., 2021; Leung et al., 2016). the global prevalence of metabolic diseases ranges from 25 to 34% depending on diagnostic criteria, with the International Diabetes Federation (IDF) definition estimating approximately 28.2% of the adult population affected. Metabolic diseases increase the risk of cardiovascular and diabetic complications, primarily resulting from the interplay of genetic and environmental factors with insulin resistance as the central mechanism (Kazankov et al., 2018; Boulangé et al., 2016). Data show that diabetes caused 3.4 million deaths worldwide in 2024, and the number of people with diabetes globally has now reached 589 million, with projections indicating it will rise to 853 million by 2050. Although numerous studies have been conducted to elucidate the targets of metabolic diseases and explore their specific mechanisms and therapies, most previous research has focused on single targets (Yang et al., 2024; Li H. Y. et al., 2023). This reductionist approach is not conducive to understanding these complex diseases and their treatments from a holistic, system-level perspective. Consequently, there is an urgent need to develop innovative therapeutic strategies targeting the multi-factorial mechanisms and underlying pathophysiological processes of metabolic diseases.

The gut microbiota is a system composed of trillions of commensal bacteria (Bao et al., 2022; Wei et al., 2024; Lai et al., 2023a). Large numbers of bacterial populations and subtle symbiotic relationships determine their complex functions in the human body (Cani et al., 2007; Carrizales-Sánchez et al., 2021). The gut microbiota is known to have functions including: digestion and absorption, synthesis of vitamins, regulation of the immune system, elimination of pathogens, removal of toxins and carcinogens, and support of the intestinal tract (Dabke et al., 2019; Demir et al., 2022; Jie et al., 2017). The interconnections and functionalities of the gut-organ axes including the gut-brain axis, gut-lung axis, and gut-liver axis, are being continuously investigated, resulting in a substantial body of novel insights (Qin et al., 2012; Qu et al., 2025; Turnbaugh et al., 2008). Gut microbiota serves as a key environmental factor in the development and progression of metabolic diseases by maintaining intestinal homeostasis and regulating systemic metabolism. Under normal conditions, gut microbiota ferments dietary fiber to produce short-chain fatty acids (SCFAs) that modulate energy metabolism, immune function, and intestinal barrier integrity (Chen Y. et al., 2025). In contrast, high-fat and high-sugar diets induce gut dysbiosis, characterized by reduced microbial diversity, depletion of SCFA-producing beneficial bacteria, and overgrowth of Gram-negative pathogenic bacteria (Bao et al., 2025). Gut dysbiosis disrupts intestinal tight junctions and increases intestinal permeability, leading to the entry of lipopolysaccharide (LPS) into the bloodstream and causing metabolic endotoxemia (Lei et al., 2025; Ao et al., 2026). LPS activates the TLR4 inflammatory pathway, triggers insulin receptor substrate phosphorylation via JNK/IKK signaling, and induces insulin resistance. Meanwhile, it activates NF-κB to release pro-inflammatory cytokines, further aggravating metabolic inflammation and insulin resistance (Zhang et al., 2026; Paththuwe Arachchi et al., 2025; Lai et al., 2025b). Additionally, disturbed microbial metabolism further impairs glucose and lipid metabolism and energy homeostasis through reduced SCFA production, elevated trimethylamine N-oxide (TMAO) generation, and abnormal bile acid metabolism (Lai et al., 2025a; Chen G. et al., 2025; Lu et al., 2026).

Ethnomedicine is a valuable treasure of the world and Chinese Medicine is an integral part of it (Zhu et al., 2023). It adopts a holistic, systems-oriented perspective on human physiology that aligns closely with modern systems biology (Xue et al., 2023; Zhao et al., 2022). Instead of targeting isolated disease molecules, TCM conceptualizes the human body as an integrated network of functional systems that sustain homeostasis through dynamic cross-talk and interactions (You et al., 2020; Zhang et al., 2022). A core principle of TCM, “bian zheng lun zhi,” highlights tailored interventions based on the pattern identification of functional dysregulations (Dong et al., 2024). Ancient canonical works such as Huangdi Neijing, Nan Jing, and Shanghan Zabing Lun systematically elaborate physiological regulation, pathological mechanisms, and preventive strategies—concepts that are now being re-evaluated and validated through network pharmacology and multi-omics approaches (Zeng et al., 2024; Huang Q. et al., 2024; Liu et al., 2022). Classical TCM formulas, including Xiao Qing Long Decoction, Xiao Chai Hu Decoction, and Si Jun Zi Decoction, have been used continuously from ancient times to the present. They exert therapeutic actions through multi-component and multi-target mechanisms, acting on complex disease-related networks rather than single signaling pathways (Sun et al., 2023; Lin et al., 2020; Gong X. et al., 2020).

Modern pharmacological research has unveiled the specific components of herbal medicine formulations and their mechanisms in treating various diseases. However, the comprehensive role of these formulations in disease prevention and treatment still necessitates exploration from multiple perspectives. Emerging technologies such as network pharmacology, high-throughput sequencing, and metabolomics have catalyzed a paradigm shift in the research of herbal medicine formulations from a singular target or component focus to a holistic systems biology approach (Dey, 2019; Nie et al., 2018). This integrated strategy is highly consistent with the multi-component, multi-target characteristics of traditional herbal medicines in various medical systems (Feng et al., 2019; Grover et al., 2025; Li Y. et al., 2021). Existing reviews focus on isolated components or single diseases, failing to capture the multi-component, multi-disease systemic perspective inherent to both herbal medicines and metabolic diseases. Accordingly, exploring how herbal formulas regulate the gut microbiota to improve metabolic diseases holds unique advantages and considerable developmental potential (Fan and Pedersen, 2020; Guo et al., 2023; Lai et al., 2022a,b).

Therefore, this review aims to delineate the intricate compositions of various traditional herbal formulas and their associated functionalities, alongside the mechanisms by which these formulations exert their therapeutic effects on metabolic diseases.

## 2 Review methodology

This review was conducted following the methodological framework of the Preferred Reporting Items for Systematic Reviews and Meta-Analyses (PRISMA) 2020 guidelines to ensure transparency and reproducibility. Systematic searches were conducted in PubMed (via NCBI) and Web of Science Core Collection (Clarivate Analytics) from 2015 to 2025. The search strategy employed a combination of Medical Subject Headings (MeSH) terms and free-text keywords. The complete search strategy for PubMed was: PubMed: (“herbal medicine”[MeSH Terms] OR “herbal medicine”[Title/Abstract] OR “traditional Chinese medicine”[Title/Abstract] OR “Chinese herbal formula”[Title/Abstract]) AND (“gut microbiota”[MeSH Terms] OR “gut microbiota”[Title/Abstract] OR “intestinal microbiota”[Title/Abstract] OR “gut flora”[Title/Abstract]) AND (“metabolic diseases”[MeSH Terms] OR “metabolic syndrome”[Title/Abstract] OR “obesity”[Title/Abstract] OR “type 2 diabetes”[Title/Abstract] OR “non-alcoholic fatty liver disease”[Title/Abstract] OR “NAFLD”[Title/Abstract] OR “T2DM”[Title/Abstract]) Web of Science Core Collection: TS = (“herbal medicine” OR “traditional Chinese medicine” OR “Chinese herbal formula”) AND TS = (“gut microbiota” OR “intestinal microbiota” OR “gut flora”) AND TS = (“metabolic diseases” OR “metabolic syndrome” OR “obesity” OR “type 2 diabetes” OR “non-alcoholic fatty liver disease” OR “NAFLD” OR “T2DM”).

All retrieved records were imported into EndNote X9 (Clarivate Analytics) for deduplication. Titles and abstracts were screened independently by two reviewers (XL and YL) against the eligibility criteria. Full-text articles were retrieved for potentially eligible records and assessed independently by the same two reviewers. The study selection process is documented in a PRISMA 2020 flow diagram (Supplementary Figure S1). Inclusion criteria comprised original research articles investigating herbal medicine-mediated gut microbiota modulation in metabolic disease animal models. Exclusion criteria included reviews without original data, non-peer-reviewed literature, and duplicate publications. Additionally, this review confirms that all evidence is based on *in vivo* animal studies with appropriate controls or *in vitro*/*ex vivo* experiments of equivalent quality. Due to substantial heterogeneity in herbal interventions, animal models, outcome measures, and methodological quality, meta-analysis was deemed inappropriate. Instead, findings were synthesized narratively and structured thematically according to: intervention type (herbal polysaccharides, non-polysaccharide components, herbal formulations), and metabolic disease category (obesity, NAFLD, T2DM). The strength of evidence for each mechanistic claim was graded based on consistency across independent studies and methodological rigor. The study selection process is documented in a PRISMA 2020 flow diagram (Supplementary Figure S1).

## 3 Interrelationships between metabolic diseases and the gut microbiota

**Figure 1:**
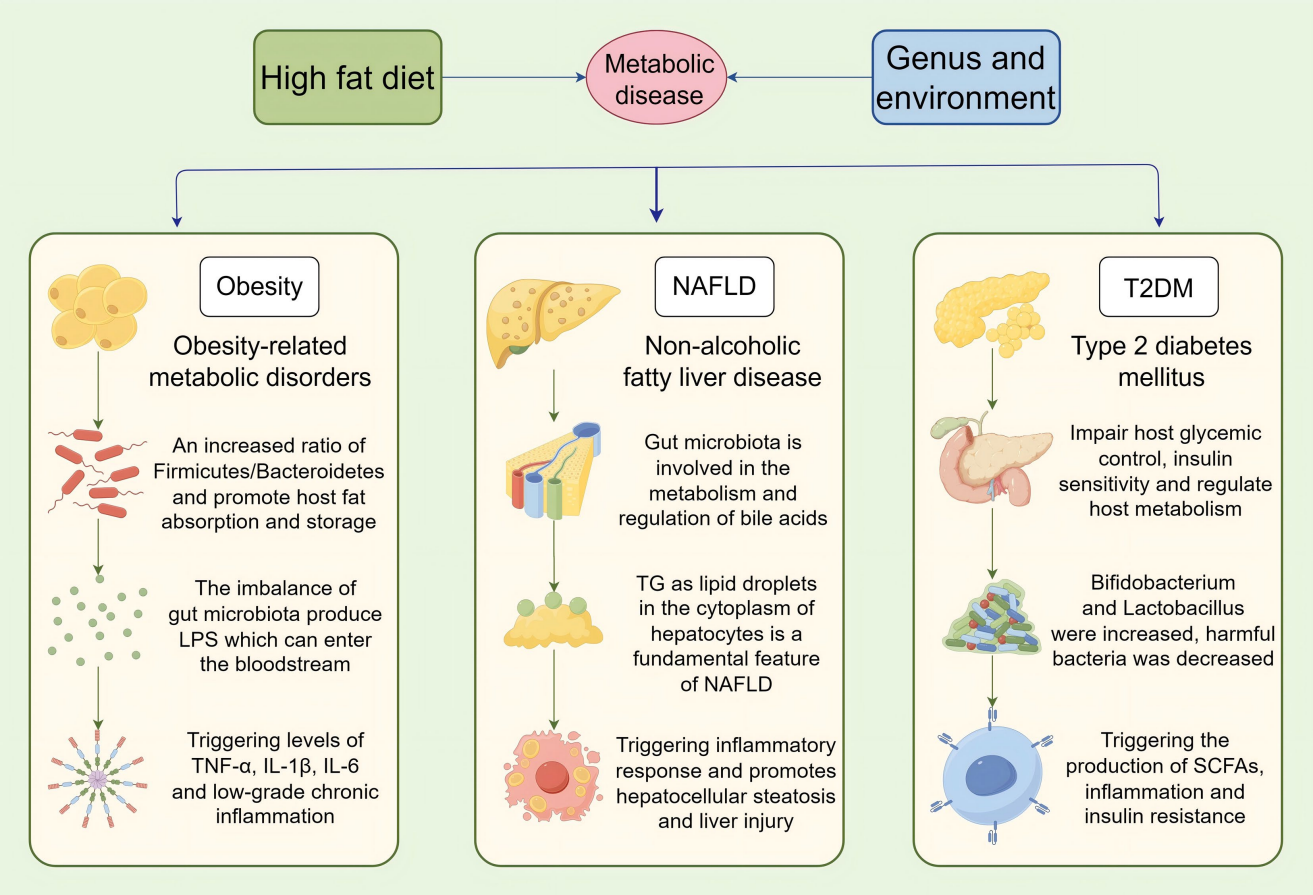
Interrelationships between metabolic diseases and gut microbiota dysbiosis. This schematic illustrates how high-fat diet (HFD) and genetic/environmental factors contribute to three major metabolic diseases through gut microbiota alterations. In obesity, increased Firmicutes/Bacteroidetes ratio promotes fat absorption; gut microbiota imbalance produces lipopolysaccharides (LPS) that trigger systemic inflammation via TNF-α, IL-1β, and IL-6. In non-alcoholic fatty liver disease (NAFLD), gut microbiota dysregulation affects bile acid metabolism and hepatic lipid accumulation; triglyceride (TG) deposition in hepatocytes triggers inflammatory responses leading to hepatocellular steatosis. In type 2 diabetes mellitus (T2DM), Bifidobacterium, Lactobacillus decrease while harmful bacteria increase, impairing glycemic control; short-chain fatty acids (SCFAs) production affects inflammation and insulin resistance. The differential emphasis on specific pathways (energy harvesting in obesity, bile acid dysregulation in NAFLD, SCFA deficiency in T2DM) provides a rationale for disease-specific therapeutic targeting while acknowledging shared microbial targets by figdraw.com.

Metabolic diseases are a multifaceted pathological condition characterized by the coexistence of multiple metabolic abnormalities in a single individual, including abdominal obesity, hyperglycemia, dyslipidemia, and hypertension (Figure 1) (Chen et al., 2023; Le Chatelier et al., 2013; Mayneris-Perxachs et al., 2021). These abnormalities, when clustered together, significantly elevate the risk of developing chronic diseases such as cardiovascular disease and type 2 diabetes mellitus (Gomes et al., 2018; Fujiwara et al., 2015). The etiology of metabolic diseases remains incompletely understood but is believed to result from the interplay of polygenic factors and various environmental influences. Genetic predisposition plays a crucial role in the development of metabolic diseases, with a familial aggregation pattern observed; offspring of affected parents are at an increased risk of developing the syndrome (Green et al., 2020). Moreover, metabolic diseases are closely associated with several environmental factors, particularly dietary habits characterized by high fat and high carbohydrate intake (Tilg and Moschen, 2014; Zhang et al., 2010). Such dietary patterns often lead to obesity, which in turn increases the risk of insulin resistance. Insulin resistance, the core pathophysiological mechanism underlying metabolic diseases, is defined by a reduced sensitivity to insulin, impairing the ability of liver, muscle, and adipose tissues to effectively utilize insulin (Ridaura et al., 2013; Zhang L. W. et al., 2024). The diagnostic criteria for metabolic diseases vary among different organizations but generally encompass central obesity, hyperglycemia, hypertension, and dyslipidemia (Li H. Y. et al., 2021). For instance, the International Diabetes Federation (IDF) defines metabolic diseases as the presence of central obesity along with any two of the following four criteria: elevated triglyceride levels, reduced high-density lipoprotein cholesterol levels, increased blood pressure, and elevated fasting blood glucose (Xiao et al., 2023). The management of metabolic diseases necessitates a comprehensive intervention approach, encompassing lifestyle modifications and pharmacological treatments. Lifestyle changes include weight reduction, increased physical activity, and dietary adjustments. Pharmacological interventions focus on controlling blood glucose, regulating lipid levels, and lowering blood pressure (Zhang X. et al., 2021). Genetic marker-based risk assessment and development of personalized therapeutic strategies for the treatment of metabolic diseases may become a hot research direction (Table 1).

**Table 1:** Herbal polysaccharide and non-polysaccharide components ameliorate metabolic diseases by modulating gut microbiota.

Herbal medicine (HM)	Component	Functions	Animal model	Gut microbiota regulation	Gut metabolites regulation	Gene and protein regulation	References
*Inonotus obliquus*	Polysaccharide	T2DM	STZ induced KM mice	IN increased the abundance of beneficial bacteria *Akkermansia* and *Lactobacillus* and decreased *Bacteroides* and *Parabacteroides*	IN promotes the production of beneficial metabolites SCFAs, regulates bile acid metabolism	IN increased the expression of tight junction proteins such as ZO-1, Ki-67, and MUC2, and decreased the production of inflammatory factors TNF-α and IL-6	Su et al. (2022)
*Plantago asiatica L.*	Polysaccharide	Obesity	High-fat diet-fed (HFD) induced Wistar rats	PLP promoted *Bacteroides vulgatus* and *Lactobacillus fermentum*, inhibited the harmful bacteria *Alistipes obesi* and *Clostridium bolteae*	PLP increased the concentration of acetic acid, propionic acid and butyric acid	PLP reduce oxidative stress, and increase the expression of intestinal tight junction proteins	Nie et al. (2019)
*Poria cocos*	Oligosaccharide	Obesity	HFD induced mice	PCOs increased the abundance of *Lactobacillus* and *Clostridium* and decreased the abundance of *Helicobacter* and *Alistipes*	PCOs increased the levels of SCFAs and UDCA, and decreases 5-HT levels.	PCOs increased the intestinal tight junction proteins ZO-1, Occludin, and Claudin-1, and inhibited the mRNA expression of TNF-alpha, IL-1β, IL-6, COX-5b, and MCP-1	Zhu et al. (2022)
*Poria cocos*	Polysaccharide	Obesity	HFD induced ob/ob mice	WIP promoted *achnospiracea, Clostridium XIVa* and *Clostridium IV,* inhibitied *Megamonas* and *Proteus*	WIP increased acetic, propionic, and butyric acids, increased UDCA levels, and decreased plasma LPS levels	WIP significantly increased SOD activity, decreased MDA levels, increased ZO-1 and Occludin, and activated the PPAR-γ signaling pathway	Sun et al. (2019)
*Unripe fruits*	Polysaccharide	Obesity	HFD induced C57BL/6 mice	RP2 increased *Lactobacillus* and *Bifidobacterium,* decreased *Olsenella* and *Ruminiclostridium_9*	RP2 increased SCFAs in feces and decreased LPS levels	RP2 decreased LPO, inhibited the TLR4/NF-κB signaling pathway and the expression of TNF-α, IL-6, and IL-1β	Huang Y. et al. (2024)
*Ganoderma lucidum*	Polysaccharide	Obesity	HFD induced C57BL/6 mice	BSLGP increased the abundance of the beneficial bacteria *Allobaculum* and *Bifidobacterium* and decreased the abundance of the harmful bacteria *Blautia* and *Ruminiclostridium_9*	BSLGP increased SCFAs in feces and decreased LPS levels	BSLGP increased ZO-1, Occludin and Claudin-5, inhibition of TLR4/NF-κB signaling pathway, and decreased TNF-α, IL-1β and MCP-1	Sang et al. (2021)
*Dendrobium officinale*	Polysaccharide	T2DM	STZ induced C57BL/6 mice	LDOPs increased the number of beneficial bacteria such as *Bifidobacterium, Lactobacillus,* and *Acromobacter*	LDOPs increased SCFAs, especially butyric acid	LDOPs promoted the secretion of glucagon-like peptide-1 (GLP-1) and peptide YY (PYY) by activating GPR41 and GPR43 receptors	Fang et al. (2022)
*Ixeris chinensis*	Triterpenoids	NAFLD	HFD induced C57BL/6 mice	IC increased the number of beneficial bacteria of the *Akkermansiaceae* and decreased the number of harmful bacteria of the *Lachnospiraceae* and *Muribaculaceae*	IC affected the pathways of glutamine and glutamate metabolism, vitamin B6 metabolism, arginine and proline metabolism	IC significantly reduced the levels of IL-6, IL-1β and TNF-α in the liver, and attenuated the hepatic inflammatory response	Jin et al. (2022)
*Ganoderma lucidum*	Water extract	Obesity	HFD induced C57BL/6 mice	WEGL decreased the Firmicutes/Bacteroidetes ratio and *Proteobacteria* levels, and increased *Bacteroidetes* and *Clostridium* species	WEGL increased the production of SCFAs, activated regulatory T cells	WEGL decreased the levels of FFA, TNF-α, IL-1β, IL-6, LPS, and TLR4, inhibited the phosphorylation of JNK and the activation of NF-κB	Chang et al. (2015)
*Siraitia grosvenorii*	Mogrosides	T2DM	STZ induced rats	SG increased the beneficial *bacterium Elusimicrobium* and decreased *deleterious bacterium Proteobacteria*	SG increased intestinal acetate, propionate, and butyrate levels, and regulated bile acid metabolism	SG decreasied the levels of pro-inflammatory factors TNF-α and IL-6, and increased anti-inflammatory factors IL-10	Zhang Y. et al. (2021)
*Quercetin*	Flavonoids	Obesity	HFD induced C57BL/6 mice	Quercetin specifically increased the abundance of *Akkermansia muciniphila*	The motabolites of indole-3-lactic acid (ILA) up-regulated 12α-hydroxylase (CYP8B1), promoted the conversion CA.	ILA inhibited fat accumulation by enhancing FTO expression, quercetin decreased TNF-α and IL-6, and increased IL-10	Liu et al. (2025)
*Ampelopsis grossedentata*	Flavonoid	T2DM	STZ induced rats	AGT increased the abundance of *bifidobacteria* and *clostridia*	AGT increased the number of BSH-activated bacteria and decreased the levels of T-β-MCA and T-α-MCA	AGT activated FXR and induced fibroblast growth factor 15 (FGF15), inhibited bile acid synthesis and gluconeogenesis	Hu et al. (2023)
*Curcumin*	Polyphenol	Obesity	HFD induced C57BL/6 mice	Cur increased *bifidobacteria* such as *Bifidobacterium, Mucinophilus, Akkermansia*, *Parabacteroides*, and *Alloprevotella*	Cur promotes the production of acetic acid, propionic acid, butyric acid, and bile acids	Cur inhibited the activation of the NF-κB signaling pathway, inhibits the phosphorylation of JNK and P38	Li S. et al. (2021)

Different metabolic diseases are often interconnected. Obesity, a risk factor for T2DM, also contributes to NAFLD (Li C. et al., 2021; Li Q. et al., 2020). The gut microbiota of obese, hyperlipidemic, NAFLD, and T2DM patients differs from that of healthy individuals, with fewer butyrate - producing bacteria and more harmful and opportunistic pathogens (Zhou et al., 2024). Functionally, their gut microbiota produces fewer SCFAs and more harmful metabolites like endotoxins and hydrogen sulfide (Wang et al., 2021). This gut microbiota imbalance is central to metabolic diseases, making modulating it a promising solution.

### 3.1 Obesity-induced metabolic disturbances and gut microbiota imbalance

Obesity is a disease that causes metabolic disorders in the human body and is closely related to gut microbiology, which is caused by the interaction of various complex factors such as genes, diet and lifestyle (Piché et al., 2020; Cox et al., 2015). According to the latest data, overweight and obesity in adults and children are showing a worrying trend worldwide, with 45.1% of the population being overweight or obese. Obesity can lead to type 2 diabetes, gout, Alzheimer’s disease and many other diseases and is associated with more than 60% of deaths (Liu et al., 2020). Gut microbiota has a huge impact on the host’s metabolism, and more and more studies continue to confirm the gut microbiota by directly participating in or regulating various metabolic processes in the organism. Yu et al. analyzed the gut microbiota of obese patients and found that compared with the gut microbiota of normal-weight populations, gut microbiota of obese patients were altered in terms of their number, proportion, and function (Yu et al., 2020). Liu et al. showed that gut microbiota are directly linked to the development of metabolic diseases such as obesity and can influence body weight and body metabolic function (Liu et al., 2021). Firstly, in obese individuals, the gut microbiota composition significantly changes, typically shown as an increased ratio of Firmicutes to Bacteroidetes, which may be closely related to the development of obesity (Vujkovic-Cvijin et al., 2020). Secondly, certain gut microbes can efficiently harvest energy from food and promote host fat absorption and storage, leading to obesity (Khan et al., 2018). Lastly, the imbalance of gut microbiota in obesity may cause low-grade chronic inflammation which produce inflammatory mediators like lipopolysaccharides (LPS). These LPS can enter the bloodstream, trigger systemic inflammatory responses, and promote the development of insulin resistance, further worsening obesity-related metabolic disorders (Khan et al., 2021). Given the close link between gut microbiota and chronic metabolic diseases, modulating their composition and structure to influence gut function and short-chain fatty acid metabolism offers a novel strategy for weight control and energy metabolism balance, providing new insights for obesity intervention.

### 3.2 Pathophysiological mechanisms of NAFLD linked to gut microbiota dysbiosis

NAFLD is a common chronic liver disease, and 80% of obese patients have varying degrees of NAFLD (Simon et al., 2021). NAFLD can lead to cirrhosis and hepatocellular carcinoma, and the risk of death rises progressively as the severity of the disease increases, with patients with NAFLD having an have a 93% increase in all-cause mortality compared to normal subjects (Younossi et al., 2017). With the gradual shift of people’s dietary habits from traditional plant-based foods to a high-fat, high-energy dietary structure, the obesity rate of the whole population is rising year by year, and the public health problem of NAFLD is becoming increasingly serious. Data from the World Health Organization shows that the global prevalence of NAFLD is about 25% of the total adult population (Simon et al., 2021). Pathologically the deposition of triglycerides (TG) as lipid droplets in the cytoplasm of hepatocytes is a fundamental feature of NAFLD, while gut microbiota dysbiosis plays an important role in the development of hepatic steatosis (Gurian et al., 2020). On the one hand, the gut microbiota is involved in the metabolism and regulation of bile acids, and the altered composition of the gut microbiota in patients with NAFLD leads to abnormal bile acid metabolism, which in turn affects hepatic fat metabolism and inflammatory response (Liu et al., 2021). On the other hand, due to the impaired intestinal barrier function, LPS produced by intestinal bacteria can enter the liver and activate the immune cells in the liver, triggering an inflammatory response that promotes hepatocellular steatosis and liver injury, exacerbating the condition of NAFLD (Xiang et al., 2023). Dietary changes are considered one of the easiest means to modulate and remodel gut microbiota, and targeting gut flora to ameliorate hepatic and intestinal diseases has great potential (Koh et al., 2016). Therefore, intervening in fatty liver by modulating gut microbiota for ameliorating NAFLD caused by the increasingly serious, high-fat dietary structure in our society is of great clinical significance and social value.

### 3.3 Gut microbiota modulation as a therapeutic target for T2DM

Diabetes mellitus, a metabolic disease marked by chronic hyperglycemia, is a major global public health challenge (Panigrahy et al., 2017; Kopf et al., 2021). The main harm of diabetes lies in its complications caused by long-term hyperglycemia, which can affect multiple organ systems, such as the cardiovascular, skeletal muscle, urinary, and nervous systems (Cerbone et al., 2021). These complications can lead to severe health issues like bacterial infections and sepsis, and may even be life-threatening (Tomkins et al., 2022). In T2DM patients, gut microbiota diversity declines, with reduced beneficial bacteria and increased harmful bacteria (Nakagami, 2019). This dysbiosis may impair host glycemic control and insulin sensitivity. Gut microbiota ferment dietary fiber to produce SCFAs, which regulate host metabolism (Kita et al., 2024). However, SCFA production may decrease in T2DM patients, further affecting glycemic control. Moreover, gut barrier dysfunction in diabetes allows bacteria and metabolites to enter the bloodstream, triggering inflammation and insulin resistance (Rea et al., 2018). Collectively, the composition and function of gut microbiota change in many ways, such as shifts in metabolic pathways, gut permeability, immune regulation, and bile acid (BAs) metabolism. These changes can directly or indirectly affect T2DM progression and offer potential therapeutic targets. Some studies have found that certain gut microbiota changes are linked to insulin resistance and chronic inflammation, which are at the core of T2DM pathology (Liang et al., 2024). Interventions like dietary changes, prebiotics, probiotics, and drugs can manipulate microbial activity to improve glucose homeostasis and reduce inflammation, showing positive therapeutic effects. Thus, further exploring the role and mechanisms of gut microbiota in T2DM and developing targeted therapies will provide new angles and methods for T2DM clinical management (Table 2).

**Table 2:** Herbal medicine formulations alleviate metabolic diseases by modulating gut microbiota and metabolites.

Herbal medicine formula (HMF)	Functions	Modern pharmacy effect	Animal model	Gut microbiota regulation	Gut metabolites regulation	Gene and protein regulation	References
Shen-Yan-Fang-Shuai formula (SYFSF)	Reducing blood glucose, triglyceride, cholesterol and anti-inflammatory	Obesity	HFD-induced C57BL/6 mice	SYFSF increased the relative abundance of *Lactobacillales* while decreased that of *Bacteroidales*	SYFSF modulates metabolites, increases short-chain fatty acid production, affects bile acid metabolism, and decreases lipopolysaccharide levels	SYFSF lowered serum concentrations of TNF-α and IL-1β, and increased the expression of tight junction protein ZO-1	Wang P. X. et al. (2020)
Jian Pi Tiao Gan Yin (JPTGY)	Soothing the liver, regulating qi, strengthening the spleen, and resolving phlegm	Obesity	HFD induced C57BL/6 mice	JPTGY induced enrichments in *Lachnospiraceae NK4A136 group, Oscillibacter, Turicibacter, Clostridium sensu stricto 1,* and *Intestinimonas*	JPTGY regulated linoleic acid (LA) metabolism paths, alpha-linolenic acid (ALA) metabolism paths, glycerophospholipid metabolism paths, arachidonic acid (AA) metabolism paths, and pyrimidine metabolism paths	JPTGY decreased the levels of total cholesterol (TC), triglyceride (TG), low-density lipoprotein cholesterol (LDL-C), and elevated high-density li poprotein cholesterol (HDL-C)	Dong et al. (2022)
Kang Shuai Lao Pian (KSLP)	Benefiting Qi and nourishing Yin, tranquilizing the mind and calming the spirit	Obesity	HFD induced C57BL/6 mice	KSLP reversed HFD-induced changes in the abundance of certain genus including *Intestinimonas, Oscillibacter, Christensenellaceae_R-7_group, Aliihoeflea* and *Ruminococcaceae_UCG-010*	KSLP reversed the contents of 22 metabolites in response to HFD treatment	*N*	Gong S. et al. (2020)
Xiao Ke Yin (XKY)	Mitigating hyperglycemia, IR, hyperlipidemia, and hepatic pathological injury	Obesity	HFD induced C57BL/KSJ-db/db mice	XKY decreased secondary bile acid producing bacteria (*Clostridia* and *Lachnospircaeae*)	XKY lowered fecal secondary bile acid [lithocholic acid (LCA) and deoxycholic acid (DCA)] levels, and regulated amino acid and tryptophan metabolism	XKY decreased IL-6 and TNF-α, and promoted hepatic BA synthesis by inhibiting the FXR FGF15 signaling pathway	Li M. et al. (2023)
Xiongdanjiuxin pill (XP)	Alleviating vascular endothelial damage, and improving liver and heart function damage	Hyperlipidemia (HLP)	HFD induced SD rat	XP reduced the relative abundance of firmicutes, while increasing the relative abundance of *bacteroidetes*	XP could significantly reduce the levels of inflammatory cytokines, and inhibit TLR4 signaling pathway, thereby reducing liver inflammation	*N*	Wu Y. J. et al. (2023)
Erchen Decoction (ECD)	Suppressing inflammation, reducing blood lipid levels, and improving insulin resistance	Obesity	HFD induced SD rat	ECD increased the abundance of SCFA-producing bacteria, including *Lactobacillus, Bifidobacterium,* and *Butyricicoccus*, and lowered the abundance of disease-related bacteria, such as *Bacteroides, Para bacteroides,* and *Sediminibacterium*.	ECD increased total SCFAs levels, especially butyric acid	ECD decreased histone deacetylase 1 expression and increased acetyl-histone 3-lysine 9 (H3K9ac) levels	Zhang L. et al. (2023)
Danggui-Shaoyao-San (DSS)	Bolstering the blood cycle and alleviating stasis	Metabolic diseases	Fructose-fed Wistar rats	DSS significant up-regulated the number of *Akkermansia*	DSS slowed down the reaction of gluconeogenesis, and reduced the blood glucose, glycine, alanine and lactate	DSS down-regulated tumornecrosis factor alpha (TNF-α) and hydroxysteroid 17β-dehydrogenase 7 (HSD17β7)	Yin et al. (2021)
Bawei Guben Huashi Jiangzhi Decoction (BGHJ)	Reducing cholesterol levels, and helping to lose weight	Obesity	HFD-induced SDO rat	BGHJ increased the relative abundances of *Dubosiella, Ligilactobacillus, Muribaculaceae-unclassified UCG-005*, and significantly lowered *Akkermansia*	BGHJ could effectively regulated Biotin metabolism, Porphyrin and chlorophyll metabolism, AA metabolism, Tryptophan metabolism, Aminoacyl-tRNA biosynthesis, Biosynthesis of unsaturated fatty acids	BGHJ significantly increased the POMC mRNA expression in the hypothalamus	Yi et al. (2024)
Shenling baizhu powde (SLBZP)	Tonifying qi and strengthening spleen, relieving dampness and Stopping diarrhea	NAFLD	HFD-induced NAFLD SD rat	SLBZP increased the relative abundance of SCFA-producing bacteria, including *Bifidobacterium* and *Anaerostipes*	*N*	SLBZP decreased the serum level of endotoxin, tumor necrosis factor α (TNF-α), interleukin-1β (IL-β) (*p* < 0.05), and decreased the expression of TLR4 pathway related protein	Zhang et al. (2018)
Qinghua Fang (QHF)	Improving liver fat, treating various metabolic disorders	NAFLD	HFD induced Wistar rat	QHF reduced the abundance of pathogens, such as *Clostridium glycyrrhizinilyticum,* and increased butyrate-producing bacteria, such as *Flintibacter butyricus* and *Blautia luti*	QHF increased butyrate, and increased blood lipid levels	QHF reduced the inflammation of liver cells, decrease the level of transaminases and improve the fatty deposition in the liver of rats with NAFLD	Wang et al. (2021)
Sheng-Jiang Powder (SJP)	Mitigating insulin resistance, regulating immune response	NAFLD	HFD induced C57BL/6 mice	SJP modulated the relative abundance of SCFAs producing bacteria, including *norank-f-Erysipelotrichaceae* and *Roseburia. Conclusions*	SJP have a impact on biosynthesis of amino acid, cysteine and methionine metabolism, glycine pathways	SJP increased the expression of PPARc mRNA and PPARc protein level, and decreased the expression of FASN	Li J. et al. (2020)
Si Miao Formula (SMF)	Alleviating inflammation and renal injury	NAFLD	HFHS diet-induced C57BL/6 mice	SMF significantly increased the proportion of *Akkermansia muciniphila*	SMF downregulated the biosynthesis of fatty acids and stimulated the insulin secretion pathway	SMF downregulated the expression of genes implicated in the metabolism of lipids (Acly, Fas, Acc, Scd-1) and pro-inflammatory cytokines (Il-1β, Nlrp-3) in the livers	Chen Y. et al. (2022)
Xie Zhuo Tiao Zhi (XZTZ)	Reducing hepatic steatosis, improving insulin resistance	NAFLD	HFD induced C57BL/6 mice	XZTZ significantly enriched the abundance of *Ileibacterium valens*	XZTZ caused a significant enrichment of the purine metabolism pathway in liver tissue metabolites, with inosine, a purine metabolite	XZTZ down-regulated the expression of NLRP3, GSDMD-N, Nek7, ASC, and Caspase-1 p20, suppressed fatty acid synthesis- and transport-associated proteins FAS, CD36 and FATP5, and up-regulated the expression of proteins related to fatty acid β-oxidation, p-AMPK, p-ACC and CPT1α	Qiu et al. (2023)
Shenzhu tiaopi Granule (STG)	Strengthen the spleen and remove the dampness	T2DM	STZ induced T2DM Goto Kakizaki (GK) rats and age-matched Wistar (W) rats	Bacteroidetes, the ratio of Firmicute/Bacteroidete, Allobaculum, and Desulfovibrionaceae were signifcantly decreased in response to the STG treatment, while Lactobacillus was enriched	STG regulates lipid metabolism by altering the bile acid metabolic pathway	STG treatment can improve glucose and lipid levels	Zhao et al. (2019)
Danggui Buxue decoction (DBD)	Promoting hematopoiesis, regulating immunity, protecting cardiac and cerebral vessels	T2DM	HFD induced T2DM Goto-Kakizaki (GK) rat	DBD improved microbial diversity, increased the abundance of *Romboutsia, Blautia,* and *Lactobacillus,* and decreased the abundance of *Allobaculum,* and *Ruminococcus torques* group	DBD regulated alanine, aspartate, and glutamate metabolic pathways by decreasing inflammation-related metabolites such as 9-OxoODE, 2,3-Dinor-6-keto-prostaglandin F1 alpha, and increasing phytoestrogenic metabolites such as enterodiol, enterolactone	DBD reduced insulin levels, increased HOMA-IS, decreased HOMA-IR, and reduced serum levels of pro-inflammatory cytokines TNF-α, IL-1β, IL-18, IL-6, and oxidative stress markers MDA and ROS	Wang W. K. et al. (2022)
Linggui Zhugan formula (LGZG)	Resolving the body fluid, strengthening the spleen inducing dampness	T2DM	HFD induced T2DM C57BL/6 mice	LGZG altered the ratio of Firmicutes/Bacteroidetes, and the relative abundance of certain bacteria, such as *Bacteroides, Lactobacillus, Oscillospira,* and *Helicobacter*	LGZG improved lipid metabolism	LGZG influenced blood glucose control, decreased blood glucose levels, and increased glucose tolerance	Wu et al. (2019)
Fufang Fanshiliu decoction (FFSLD)	Strengthening the spleen, drying dampness, draining turbidity and clearing heat	T2DM	STZ induced T2DM SD rat	FFSLD increased *Lactobacillus, Akkermansia*, and Proteus, and reduced *Alistipes, Desulfovibrio,* and *Helicobacter*	FFSLD increased the metabolic pathways such as chemoheterotrophic, fermentative, and nitrate-reducing pathways	FFSLD ameliorated the abnormal levels of IL-1β, IL-6, TNF-α, and TGF-β	Li L. et al. (2022)
Ge Gen Jiao Tai Wan (GGJTW)	Transporting the heart and kidneys, clearing the mind and calming the spirit	T2DM	STZ induced T2DM SD rat	GGJTW regulates the composition of the gut microbiota and upregulate the diabetic beneficial phylum Firmicutes and bile-acid-related genus *Lactobacillus*	GGJTW upregulated expression of the bile acid receptors FXR and TGR5 and increased concentrations of GLP-1	GGJTW increased primary bile acids in colon contents	Chen et al. (2021)
Shouhuitongbian (SHTB)	Eliminating turbidity and laxative, nourishing yin and benefiting qi	T2DM	HFD induced T2DM db/db mice	SHTB significantly increased the proportions of *Bifidobacterium, Taizéra* and *Synechococcus* while decreasing the proportions of *Streptococcus* and *Lachnospiraceae_NK4A136_group*	SHTB enhanced the intestinal production of short-chain fatty acids (SCFAs) and branched short-chain fatty acids (BSCFAs)	SHTB increased mRNA expression of PI3K and Akt, and improved IR through the BCAAs-mediated mTORC1/IRS-1/PI3K/AKT signaling pathway	Wang T. et al. (2024)
Simiao Wan (SMW)	Clearing heat and removing dampness, relieving paralysis	T2DM	STZ induced T2DM C57BL/6 mice	SMW enriched in the bacteria *Allobaculum, Clostridium, Akkermansia, Lactobacilus* and *Bilophila whereas decreased Coprococcus* and *Halomonas*	SMW modulated the profiles of BAs, indicated by the reduction of conjugated BAs and 12α-OH/non-12α-OH BAs ratio in liver as well as the increase of primary BAs in feces	SMW activated farnesoid X receptor and inhibited sterol regulatory element-binding protein-1 expression	Han et al. (2021)
Shenqi formula (SQ)	Clearing heat and promoting generation of body fluid, promoting blood circulation and removing blood stasis	T2DM	HFD induced T2DM SD rat	SQ beneficially modulated the gut microbiota by increasing populations of beneficial bacteria, such as *Lachnospiraceae_NK4A136_group* and *Akkermansia,* while inhibiting harmful strains such as *Ruminococcus* and *Phascolarctobacterium*	SQ corrected disturbances in Testosterone enanthate and Glycerophospholipid metabolism	SQ interventions significantly reduced the serum levels of HDL-C, LDL-C, TC, TG, and plasma levels of TNF-α	Gao et al. (2024)
PuRenDan (PRD)	Nourishing yin and clearing heat, promoting body fluids, and relieving thirst	T2DM	STZ induced T2DM SD rats	The gut microbiota most closely related to PRD were *Prevotella* sp. *10 (H), Parabacteroides* sp. *SN4, Flavobacteriales bacterium, Bacteroides massiliensis, Alistipes indistinctus,* and *Rumi nococcus flavefaciens*	PRD regulated the levels of gut microbiota metabolites including pantothenic acid, 1-Methylhistamine, and 1-Methylhistidine	*N*	Ma et al. (2024)
Yu–Ye Tang (YYT)	Benefiting Qi, nourishing Yin, generating fluids and quenching thirst	T2DM	STZ induced T2DM SD rat	YYT increased the abundance of *Lactobacillus, Candidatus_Saccharimonas, UCG-005, Bacteroides* and *Blautia while decreased Allobaculum* and *Desulfovibrio*	YYT regulated arachidonic acid, alanine, aspartate and glutamate, arginine and proline, glycerophospholipid, pentose and glucuronate interconversions, steroid hormone, phenylalanine, terpenoid backbone biosynthesis, tryptophan, and tyrosine metabolism	YYT significantly decreased the levels of IL-6, IL-1b, and TNF-a, increased SOD and GSH-PX levels and decreased MDA levels	Ma et al. (2023)
Shenlian decoction (SL)	Treating deficiency heat in the spleen and stomach	T2DM	HFD induced T2DM C57BL/KsJ db/db mice	SL decoction could reduce the *Prevotellaceae, Rikenellaceae,* and *Helicobacteraceae,* and upregulate the abundance of *Bacteroidaceae*	SL contributed to the metabolism of starch and sucrose as well as pentose glucuronate interconversions	SL influenced lipopolysaccharide biosynthesis, riboflavin metabolism, and peroxisome	Sun et al. (2022)
LLKL	Treating inflammation, cardiovascular disease and various metabolic diseases.	T2DM	Zucker diabetic fatty rats	LLKL treatment reduced the Firmicutes/Bacteroidetes ratio, increased the numbers of *Proteobacteria* and *Actinobacteria*	LLKL enhanced the insulin signaling pathway and inhibited glycerolipid metabolism and fatty acid metabolism	LLKL treatment decreased the expressions of TLR4, MyD88 and CTSK, and decreased the LPS, TNF-α and IL-6 levels	Li M. et al. (2020)
Huangqi Guizhi Wuwu Decoction (HGWD)	Benefiting Qi, warming menstruation, harmonizing Blood and promoting paralysis	T2DM	BKS Cg-m+/+Leprdb/J (db/db) mice with DPN	HGWD regulated *Lactobacillus, Alloprevotella, Bacteroides,* and *Desulfovibio* with four metabolic pathways	HGWD remarkably regulated the unusual levels of 37 metabolites involved in sphingolipid metabolism, biosynthesis of unsaturated fatty acids, arachidonic acid metabolism, and amino acid biosynthesis pathways	HGWD increased GSH levels and SOD activity while decreasing the MDA level, HGWD reduced TNF-α, IL-1β, and IL-6 levels while raising IL-10 levels	Zhang K. et al. (2024)
Gegen Qinlian Decoction (GQD)	Relieve fever and stop diarrhea	T2DM	HFD induced T2DM GK rat	GQD enriched many butyrate-producing bacteria, including *Faecalibacterium* and *Roseburia*	GQD elevated the levels of 50 short-chain fatty acids in rat feces	GQD reduced the serum proinflammatory cytokines and expression of immune-related genes, including Nfkb1, Stat1, and Ifnrg1	Xu et al. (2020)
Gegen Qinlian Decoction (GQD)	Treating gastrointestinal diseases such as enteritis and bacillary dysentery	T2DM	STZ induced T1DM Wistar rat	GQD increased the levels of beneficial bacteria such as *Flavonifractor* and *Acetatifactor,* whereas *Butyricimonas, Anaerofustis, Butyricicoccus,* and *Gammaproteobacteria* were decreased	*N*	GQD decreased the levels of inflammatory cytokines, and increased the levels of tight junction proteins	Tian et al. (2021)

## 4 Therapeutic effects of various components of herbal medicine

**Figure 2:**
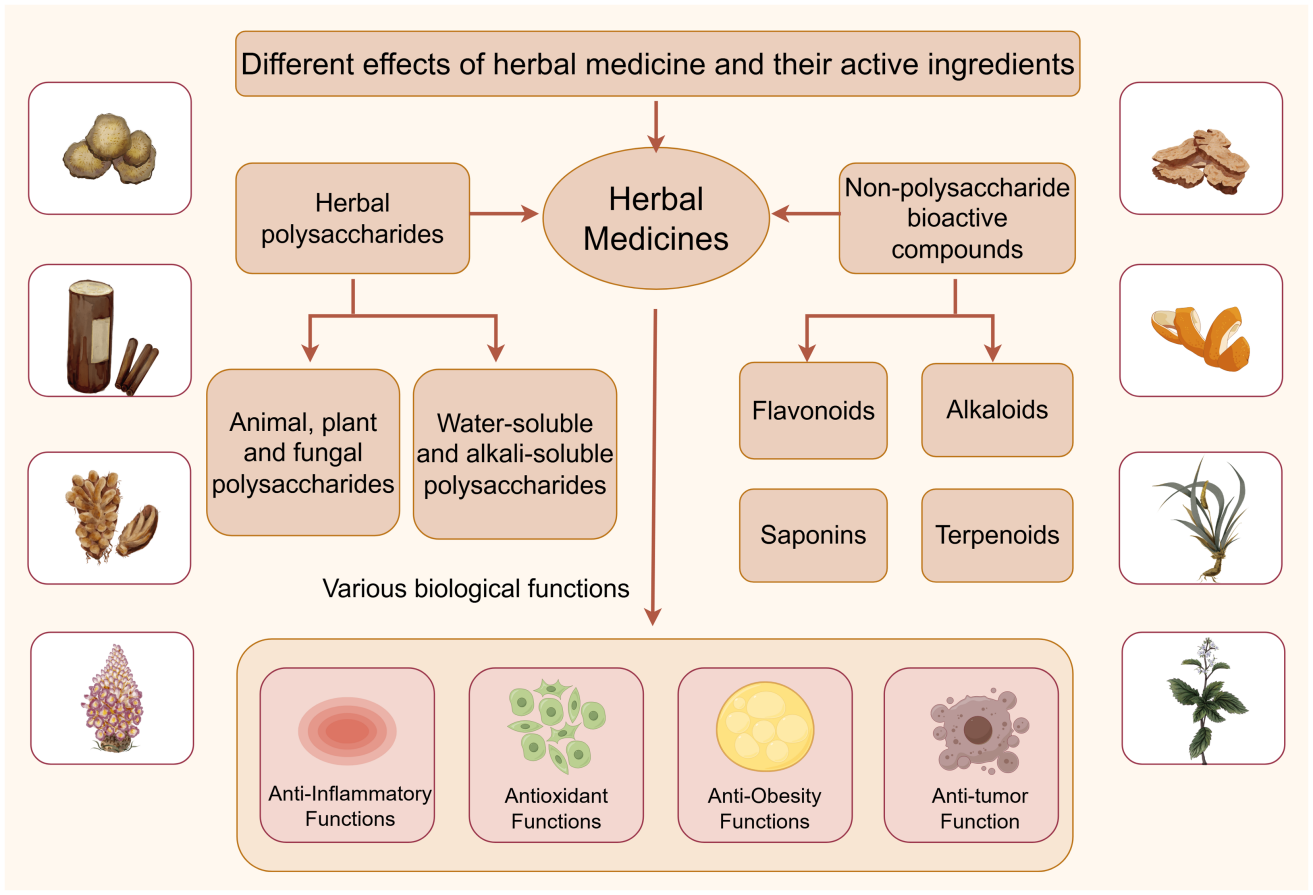
Classification of herbal medicines and their bioactive components with biological functions. This diagram categorizes herbal medicines into two major groups: Herbal polysaccharides, including animal, plant, and fungal-derived polysaccharides, as well as water-soluble and alkali-soluble polysaccharides. Non-polysaccharide bioactive compounds, comprising flavonoids, alkaloids, saponins, and terpenoids. Both categories exert diverse biological functions including anti-inflammatory, antioxidant, anti-obesity, and anti-tumor activities through multi-target mechanisms by figdraw.com.

Traditional Chinese herbs have been used in China for over 5,000 years. Bioactive compounds extracted from TCM, such as polysaccharides, alkaloids, and flavonoids, have been proven to possess various therapeutic effects, including anti-inflammatory and anti-obesity properties (Figure 2) (Lai et al., 2025a; Yang et al., 2023b; Lai et al., 2024). These bioactive compounds can modulate the gut microbiota composition to improve gut microbiota balance. Their metabolites can activate the AMPK signaling pathway and regulate insulin sensitivity, thereby ameliorating metabolic diseases (Zheng et al., 2020; Fang et al., 2023). Among them, herbal polysaccharides, serving as the main energy and nutrient source for gut microbiota, have prebiotic effects, promoting the proliferation of beneficial bacteria and inhibiting the growth of harmful bacteria (Lai et al., 2023a; Luo et al., 2022b). The indigestibility and stability of polysaccharides enable them to reach the gut and be fermented by microbiota, producing SCFAs (Luo et al., 2022a; Lai et al., 2023b). These metabolites play important roles in regulating glucose metabolism and improving insulin sensitivity (Wang Y. et al., 2024). In contrast, non-polysaccharide bioactive compounds, such as flavonoids, alkaloids, and saponins, are characterized by their small molecular weight and diverse chemical structures (Wang R. et al., 2022). Their metabolic processes often rely on microbial enzymes in the gut microbiota, such as β-glucosidase and nitroreductase, which can convert non-polysaccharides into metabolites with higher bioactivity (Song et al., 2022).

### 4.1 Mechanistic insights into the gut microbiota modulation by herbal polysaccharides

In recent years, an increasing number of studies have demonstrated that polysaccharides from herbal medicine and their metabolites play a significant role in modulating gut microbiota balance and improving metabolic diseases (Yang et al., 2023a; Wang et al., 2016; Fu et al., 2020). For instance, *Inonotus obliquus* polysaccharide (IN) maintains intestinal microecological balance by increasing the abundance of beneficial bacteria *Akkermansia* and *Lactobacillus* and decreasing *Parabacteroides* and *Bacteroides*. At the same time, IN promotes the production of beneficial metabolites such as SCFAs, regulates bile acid metabolism, increases the expression of tight junction proteins such as ZO-1, Ki-67, and MUC2, and decreases the production of inflammatory factors TNF-α and IL-6 to improve the chronic low-grade inflammatory state. These mechanisms work together to effectively reduce blood glucose levels, improve lipid metabolism, and enhance immune function, providing new ideas for the treatment of T2DM (Su et al., 2022). Another study showed that *Plantago asiatica L.* polysaccharides (PLP) promoted *Bacteroides vulgatus* and *Lactobacillus fermentum*, inhibited the harmful bacteria *Alistipes obesi* and *Clostridium bolteae*, regulated lipid metabolism, reduce oxidative stress, and increase the expression of intestinal tight junction proteins and decrease intestinal permeability in Wistar rats on a high-fat diet (Nie et al., 2019). *Poria cocos*-derived oligosaccharides (PCOs) also exhibit favorable prebiotic effects, with multi-pathway synergy to improve disorders of glucolipid metabolism. PCOs increase the abundance and concentration of beneficial bacteria such as *Lactobacillus* and *Clostridium* in the gut, raising the levels of acetate, propionate, butyrate and bile acids (UDCA), while decreasing 5-HT levels. Additionally, PCOs significantly enhance total antioxidant capacity, reduce malondialdehyde levels, increase the expression of intestinal tight junction proteins (ZO-1, Occludin, and Claudin-1), and inhibit the mRNA expression of pro-inflammatory factors (TNF-α, IL-1β, IL-6, COX-5b, and MCP-1) (Zhu et al., 2022). Interestingly, water insoluble polysaccharides (WIP) derived from *Poria cocos* can also effectively improve obesity symptoms by promoting the growth of beneficial bacteria *Lachnospiracea, Clostridium XIVa* and *Clostridium IV*, inhibiting the growth of harmful bacteria *Megamonas* and *Proteus*, increasing the expression of intestinal tight junction proteins ZO-1 and Occludin, and activating PPAR-γ signaling pathway (Sun et al., 2019). Furthermore, Huang et al. found that unripe fruits polysaccharide (RP2) increased *Lactobacillus* and *Bifidobacterium*, decreased *Olsenella* and *Ruminiclostridium_9*, and significantly increased superoxide dismutase (SOD) and glutathione (GSH) activities in the liver, decreasing malondialdehyde (LPO) levels, inhibiting the TLR4/NF-κB signaling pathway, and decreasing the expression of the pro-inflammatory factors TNF-α, IL-6, and IL-1β, thereby effectively improving obesity symptoms (Huang Y. et al., 2024). Sang et al. found that *Ganoderma lucidum* sporoderm-broken spore polysaccharide (BSGLP) can increase the abundance of beneficial bacteria like *Allobaculum* and *Bifidobacterium*, while decreasing harmful bacteria such as *Blautia* and *Ruminiclostridium_9*. BSGLP also inhibits the TLR4/NF-κB signaling pathway and reduces expression of pro-inflammatory cytokines TNF-α, IL-1β, and MCP-1, thereby alleviating obesity symptoms (Sang et al., 2021). *Dendrobium officinale* polysaccharides (PDOPs) decrease the ratio of Firmicutes to Bacteroidetes, increase the number of *Bifidobacteria, Lactobacillus,* and *Actinobacteria*, decrease the number of harmful bacteria such as *Aspergillus*, and, by activating GPR41 and GPR43 receptors, promote glucagon like peptide-1 (GLP-1) and peptide YY (PYY) secretion, improving insulin resistance and lowering blood glucose levels. These studies indicate that polysaccharides from TCM and their metabolites modulate gut microbiota balance, improve gut barrier function, and reduce inflammatory responses through multiple mechanisms (Fang et al., 2022).

### 4.2 Health benefits of non-polysaccharide phytochemicals via gut microbiota regulation

Similarly, non-polysaccharide components (triterpenoids, steroids, glycosides, volatile oils, flavonoids, and phenolic compounds) derived from various medicinal plants have received much attention for their potential to modulate the gut microbiota and ameliorate metabolic disorders (Cui et al., 2018; Zhong et al., 2023; Liu et al., 2021). For instance, Triterpenoids derived from *Ixeris chinensis* (IC) were able to increase the number of beneficial bacteria of the *Akkermansiaceae* family and decrease the number of harmful bacteria of the *Lachnospiraceae* and *Muribaculaceae* families in the intestine. Meanwhile, IC affected the pathways of glutamine and glutamate metabolism, vitamin B6 metabolism, arginine and proline metabolism, and significantly reduced the levels of IL-6, IL-1β, and TNF-α in the liver, and attenuated the hepatic inflammatory response, thus exerting a hepatoprotective effect on HFD induced NAFLD in mice. Similarly, *Ganoderma lucidum* has been extensively studied for its health benefits (Jin et al., 2022). Chang et al. found that a water extract of *Ganoderma lucidum mycelium* (WEGL) decreased the Firmicutes/Bacteroidetes ratio and *Proteobacteria* levels, and increased *Bacteroidetes* and *Clostridium* species in obese mice. WEGL also exerted anti-obesity effects by increasing the production of SCFAs, activating regulatory T cells, decreasing the levels of FFA, TNF-α, IL-1β, IL-6, LPS, and TLR4, inhibiting the phosphorylation of JNK and the activation of NF-κB, and ameliorating chronic inflammation and insulin resistance (Chang et al., 2015).

Another notable example is *Siraitia grosvenorii* (SG), whose fruit contains mogrosides that regulate gut microbiota in T2DM rats. This regulation is characterized by an increased relative abundance of the beneficial bacterium *Elusimicrobium* and a decreased relative abundance of the deleterious *Proteobacteria*. Moreover, SG significantly increases intestinal levels of acetate, propionate, and butyrate. These SCFAs stimulate the secretion of the gut hormone glucagon-like peptide-1 (GLP-1) through the activation of G-protein-coupled receptors and regulate bile acid metabolism, thereby affecting the farnesol X receptor (FXR) signaling pathway. This, in turn, regulates glucose homeostasis and insulin sensitivity. Quercetin, a flavonoid compound, has also demonstrated promising effects in modulating gut microbiota (Zhang Y. et al., 2021). Liu et al. found that quercetin specifically increased the abundance of *Akkermansia muciniphila*, and its metabolite indole-3-lactic acid (ILA) up-regulated the expression of 12α-hydroxylase (CYP8B1), facilitated the conversion of cholesterol to cholic acid (CA), and activated FXR to inhibit fat accumulation, which in turn improved obesity (Liu et al., 2025). Moreover, *Ampelopsis grossedentata* (AGT) increased the abundance of *bifidobacteria* and *clostridia*, restored gut microbiota balance, and indirectly activated FXR by increasing the number of BSH-activated bacteria and decreasing the levels of T-β-MCA and T-α-MCA. In addition, FXR activation induced the expression of fibroblast growth factor 15 (FGF15), inhibited liver bile acid synthesis and gluconeogenesis, reducing chronic inflammatory state and improving T2DM (Hu et al., 2023). Lastly, Curcumin (Cur) is a natural polyphenol compound with anti-inflammatory, antioxidant, and anti-obesity properties. Cur is able to increase the number of *Bifidobacterium, Akkermansia, Parabacteroides,* and *Alloprevotella*. In addition, Cur promotes the production of acetic acid, propionic acid, butyric acid, and bile acids, regulates the MAPK signaling pathway by inhibiting the activation of the NF-κB signaling pathway, inhibits the phosphorylation of JNK and P38, and thus effectively ameliorates obesity and its associated metabolic disorders (Li S. et al., 2021) (Table 3).

**Table 3:** Summary of outcomes.

Herbal medicine/formula		Disease model	Key microbiota changes	Metabolic outcomes	Inflammation	Evidence strength
Polysaccharides	*Inonotus obliquus* (IN)	T2DM (STZ-KM mice)	↑*Akkermansia*, ↑*Lactobacillus*	↓Blood glucose, ↓lipids	↓TNF-α, ↓IL-6	Moderate
	*Plantago asiatica* (PLP)	Obesity (HFD-Wistar rats)	↑*B. vulgatus*, ↑*L. fermentum*	↓Oxidative stress	↑Tight junction proteins	Moderate
	*Poria cocos* (PCOs)	Obesity (HFD-mice)	↑*Lactobacillus*, ↑*Clostridium*	↑SCFAs, ↑UDCA	↓TNF-α, ↓IL-1β, ↓IL-6	Moderate
	*Ganoderma lucidum* (BSGLP)	Obesity (HFD-C57BL/6)	↑*Allobaculum*, ↑*Bifidobacterium*	↓LPS	↓TNF-α, ↓IL-1β, ↓MCP-1	Moderate
Non-polysaccharides	*Siraitia grosvenorii* SG	T2DM (STZ-rats)	↑*Elusimicrobium*, ↓*Proteobacteria*	↑Acetate/propionate/butyrate	↓TNF-α, ↓IL-6, ↑IL-10	Moderate
	Quercetin	Obesity (HFD-C57BL/6)	↑*A. muciniphila*	↓Fat accumulation	↓TNF-α, ↓IL-6, ↑IL-10	Preliminary
	Curcumin	Obesity (HFD-C57BL/6)	↑*Bifidobacterium*, ↑*Akkermansia*	↑SCFAs, ↑BAs	↓NF-κB, ↓JNK, ↓P38	Moderate
Formulations	Shenling Baizhu Powder	NAFLD (HFD-SD rats)	↑*Bifidobacterium*, ↑*Anaerostipes*	↓Endotoxin	↓TNF-α, ↓IL-1β	Moderate
	Gegen Qinlian Decoction	T2DM (HFD-GK rats)	↑*Faecalibacterium*, ↑*Roseburia*	↑Butyrate	↓Nfkb1, ↓Stat1, ↓Ifngr1	Moderate
	GeGen JiaoTai Wan	T2DM (STZ-SD rats)	↑*Lactobacillus*, ↑*Akkermansia*	↑BAs (TCA, GCA), ↑GLP-1	–	Preliminary

In summary, these findings underscore the potential of natural products from medicinal plants to modulate gut microbiota and improve metabolic health (Xiao et al., 2019; Feng et al., 2025). Further research should focus on elucidating the underlying mechanisms and exploring the clinical applications of these compounds in the management of metabolic diseases.

## 5 Herbal medicine formulations improve metabolic diseases by regulating gut microbiota

**Figure 3:**
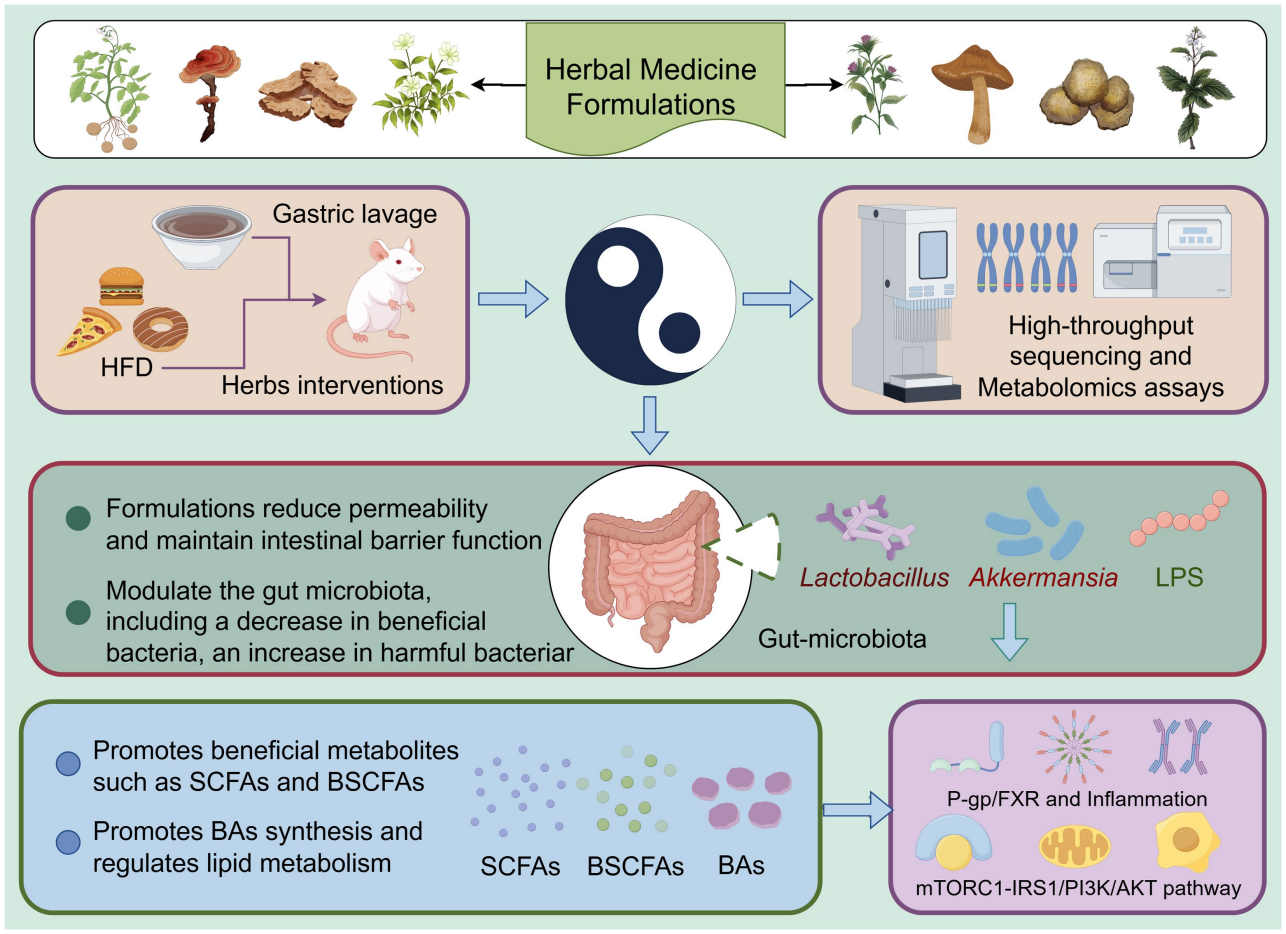
Schematic workflow of herbal medicine formulations in ameliorating metabolic diseases via gut microbiota modulation. Experimental design: HFD-induced metabolic disease models receive herbal interventions through gastric lavage, followed by high-throughput sequencing and metabolomics analyses. Gut microbiota modulation: Formulations reduce intestinal permeability and maintain barrier function by increasing beneficial bacteria (Lactobacillus, Akkermansia) and decreasing harmful bacteria and LPS levels. Metabolite regulation: Herbal formulations promote beneficial metabolites including SCFAs, branched-chain short-chain fatty acids (BSCFAs), and bile acids (BAs). Downstream signaling: These metabolites activate multiple pathways including P-glycoprotein (P-gp)/farnesoid X receptor (FXR), attenuate inflammation, and modulate the mTORC1-mediated IRS1/PI3K/AKT signaling pathway to improve insulin sensitivity and metabolic homeostasis. The downstream signaling convergence on IRS1/PI3K/AKT and FXR pathways suggests that despite compositional heterogeneity in herbal preparations, their metabolic benefits may converge on a limited set of conserved host pathways by figdraw.com.

Unlike modern pharmaceutical research focusing on chemical drugs and biologics, herbal medicines and formulas often lack the identification of specific bioactive components (Figure 3) (Bi et al., 2021; Shao et al., 2021). TCM advocates a holistic view, considering the human body as an integrated organism (Chen J. et al., 2022). The viscera and other organs are physiologically interconnected and collectively maintain the body’s homeostasis (Li S. et al., 2023). Normal physiological functions depend not only on the proper functioning of each organ and tissue but also on their complementary and antagonistic interactions to maintain physiological balance (Li C. X. et al., 2023; Nie et al., 2023). The therapeutic spectrum of these formulas interventions encompasses, but is not confined to the gut-brain, gut-lung, gut-liver, gut-heart, and gut-kidney axes, highlighting the multifaceted potential of herbs in addressing complex physiological and pathological conditions (Mu et al., 2021; Li N. et al., 2022; Hu et al., 2024).

### 5.1 Herbal medicine formulations improve obesity

Herbal medicine has garnered significant attention for its potential to ameliorate obesity through the regulation of intestinal microbiota (Fang et al., 2014). One such traditional herbal medicine formula, Shen-Yan-Fang-Shuai formula (SYFSF) can improve obesity by regulating intestinal microorganisms, metabolites and key factors. On the one hand, SYFSF reverses the imbalance of intestinal flora caused by high-fat diet by affecting the abundance of specific genera such as *Bifidobacterium, Taizelia, Anabaena,* and *Streptococcus* (Wang Z. et al., 2020). Similarly, Jian Pi Tiao Gan Yin (JPTGY) restores intestinal microbial diversity by promoting *Lachnospiraceae NK4A136, Oscillibacter, Turicibacter* and inhibiting harmful bacteria *Proteobacteria*, and influences linoleic acid, α-linolenic acid and glycerol metabolic pathways. Phospholipid metabolic pathways, improve lipid indices, and regulate intestinal microbial function, thereby improving hyperlipidemia and obesity. The comprehensive mechanism of intestinal flora and related metabolite regulation reflects the potential advantages of TCM compound in the treatment of obesity, and provides new ideas and theoretical basis for the development of new weight loss drugs and strategies (Dong et al., 2022). Another notable TCM formulation, Kang Shuai Lao Pian (KSLP), has demonstrated its efficacy in improving obesity, intestinal ecological dysregulation, and fecal metabolic disorders. KSLP improves obesity, intestinal ecological dysregulation and fecal metabolic disorders by increasing the abundance of intestinal organisms such as *Intestinimonas, Oscillibacter,* and *Christensenellaceae_R-7_group*, decreasing the number of *Proteobacteria*, regulating metabolites such as SCFAs and improving lipid indices (Gong S. et al., 2020). Meanwhile, Xiao Ke Yin (XKY) altered intestinal metabolites by increasing the abundance of beneficial bacteria such as *Bacilli, Lactobacillaceae,* and *Lactobacillus* in the intestinal tract and decreasing harmful bacteria such as *Clostridia, Lachnospiraceae, Tannerellaceae, Parabacteroides*, and *Blautia*, decreasing levels of secondary bile acids (DCA and LCA), modulating amino acid metabolic pathways to alter intestinal metabolites, and ameliorating dysfunctional glucose-lipid metabolism by improving glucose and insulin resistance (Li M. et al., 2023). Xiongdanjiuxin pill (XP) improves Hyperlipidemia (HLP) by altering gut microbial composition, increasing gut microbial diversity, decreasing endotoxin levels, regulating lipid metabolism-related gene expression, inhibiting inflammatory factors and blocking the TLR4 signaling pathway (Wu Y. J. et al., 2023).

Furthermore, Erchen Decoction (ECD) and Danggui-Shaoyao-San (DSS) are traditional Chinese herbal compound that has been shown to be protective against obesity. ECD increases the content of butyric acid, by modulating the composition and structure of gut microorganisms, which in turn activates the PPARα signaling pathway and promotes hepatic fatty acid β-oxidation through butyric acid-mediated inhibition of HDAC1 and an increase in H3K9ac, to improve obesity-related hepatic steatosis (Zhang L. et al., 2023). Danggui-Shaoyao-San (DSS) improved intestinal health in metabolic diseases rats, as evidenced by DSS increasing the abundance of the beneficial bacterium *Akkermansia* and decreasing the abundance of the potentially deleterious bacterium *Erysipelotrichaceae*, as well as decreasing the level of endotoxin, modulating the expression of genes related to lipid metabolism, inhibiting the release of the inflammatory factor TNF-α release, and alleviated metabolic diseases by modulating glucose-related metabolites and gene expression to slow down gluconeogenesis (Yin et al., 2021). Lastly, Bawei Guben Huashi Jiangzhi Decoction (BGHJ) increases the relative abundances of beneficial microbial taxa, such as *Dubosiella*, *Ligilactobacillus*, and *Muribaculaceae-unclassified UCG-005*, while notably reducing the abundance of *Akkermansia*. BGHJ also effectively regulates key metabolic pathways, including biotin metabolism, porphyrin and chlorophyll metabolism, amino acid metabolism, tryptophan metabolism, aminoacyl-tRNA biosynthesis, and the biosynthesis of unsaturated fatty acids thereby exerting a beneficial effect on spleen-deficient obesity (Yi et al., 2024). This comprehensive mechanism of action underscores the potential of TCM in addressing hyperlipidemia through gut microbiota modulation, further highlighting the multifaceted therapeutic potential of herbal medicine in metabolic disorders.

### 5.2 Herbal medicine formulations improve NAFLD

The intricate relationship between traditional herbal medicine and gut microbiota has been increasingly recognized in the context of NAFLD. Shenling baizhu powder (SLBZP) has demonstrated its potential in modulating gut microbiota by enhancing the abundance of beneficial bacteria such as bifidobacteria and anaerobes. These bacteria produce SCFAs that bind to G-protein-coupled receptors, thereby enhancing intestinal barrier function. Moreover, SLBZP alleviates NAFLD by decreasing endotoxin levels, reducing inflammation through the TLR4 signaling pathway, and down-regulating pro-inflammatory cytokines such as TNF-α and IL-1β, which in turn reduce liver inflammation and steatosis (Zhang et al., 2018). In one study, Qinghua Fang (QHF) alleviates NAFLD by increasing the abundance of *bifidobacteria and Lactobacillus acidophilus*, modulating short-chain fatty acid and bile acid metabolism, reducing endotoxin levels, inhibiting NLRP3 inflammasome activation and decreasing the release of inflammatory factors, and reducing adipose accumulation (Wang et al., 2021). Sheng-Jiang Powder (SJP) improves liver function and lipid metabolism by promoting the growth of SCFAs-producing bacteria *Bifidobacterium bifidum* and *Lactobacillus acidophilus*, lowering serum levels of TG, AST, and ALT, and regulating the expression of lipid metabolism-related genes PPARc and FASN, which affects the synthesis and metabolism of hepatic fat. This suggests that different TCM formulations may share common mechanisms in improving NAFLD through gut microbiota modulation, while also having their unique pathways (Li J. et al., 2020). Another example is Si Miao Formula (SMF), which reduced the diversity of gut microorganisms in mice fed a high-fat diet, increased the number of *Akkermansia muciniphila, Bifidobacterium,* and *Faecalibaculum*, and improved intestinal health and alleviated NAFLD by modulating bile acid metabolism and inhibiting activation of NLRP3 inflammasome, and reducing translocation of gut bacteria and their metabolites to the liver. This further highlights the multifaceted mechanisms of TCM in targeting both gut microbiota and lipid metabolism to combat NAFLD (Chen Y. et al., 2022). In a related study, Xie Zhuo Tangzhi Fang (XZTZ) significantly enriched purine metabolism pathway metabolites such as hypoxanthine and adenosine in liver tissues. This was achieved by increasing the abundance of certain beneficial bacteria such as *Ileibacterium valens, Bifidobacterium pseudologum,* and *Akkermansia muciniphila*, while decreasing the abundance of others like Faecalibacterium rodentium and *Lactobacillus reuteri*, thereby significantly altering the gut microbial community structure in NAFLD mice. Moreover, XZTZ down-regulated the expression of hepatic pyroptosis-associated proteins, such as NLRP3, GSDMD-N, Nek7, ASC, and Caspase-1 p20, and suppressed fatty acid synthesis- and transport-associated proteins such as FAS, CD36, and FATP5. It also up-regulated the expression of proteins related to fatty acid β-oxidation, such as p-AMPK, p-ACC, and CPT1α, which collectively attenuated hepatic inflammation and lipid metabolism. This comprehensive mechanism of action underscores the potential of herbal medicine in targeting multiple pathways to improve NAFLD through gut microbiota modulation (Qiu et al., 2023).

### 5.3 Herbal medicine formulations improve T2DM

#### 5.3.1 Improvement of bacterial community regulation in T2DM

Recent studies have highlighted the potential of herbal medicine formulations in modulating gut microbiota to improve metabolic health in T2DM (Wu et al., 2024; Di et al., 2022; Gao et al., 2021). For instance, Shenzhu tiaopi Granule (STG) regulates energy metabolism and lipid metabolism and indirectly improves insulin resistance by modulating the structure of the intestinal microbiota, increasing the abundance of *Lactobacillus* and decreasing the abundance of *Desulfovibrionaceae*, altering the production of SCFAs and bile acids, and regulating energy metabolism and lipid metabolism (Zhao et al., 2019). In T2DM GK rats, Danggui Buxue decoction (DBD) significantly increased the abundance of *Romboutsia, Blautia,* and *Lactobacillus*, and decreased the abundance of *Allobaculum,* and *Ruminococcus torques group*, thereby restoring the balance of the gut microbiota. In addition, DBD significantly altered the levels of a variety of metabolites, including decreasing inflammation-related metabolites such as 9-OxoODE, 2,3-Dinor-6-keto-prostaglandin F1 alpha, and increasing phytoestrogenic metabolites such as enterodiol, enterolactone, which significantly improved insulin resistance, and reduced serum levels of pro-inflammatory cytokines and oxidative stress markers MDA and ROS (Wang W. K. et al., 2022). Linggui Zhugan Formula (LGZG) modulates the structure of gut microbial communities by adjusting the structure of gut microbial communities, such as decreasing the ratio of Firmicutes/Bacteroidetes, increasing the abundance of *Lactobacillus* and *Bacteroides*, and decreasing the abundance of *Oscillospira* and *Helicobacter,* and regulating insulin, glucagon and HOMA-IR, which are the key factors, thereby improving glycolipid levels and insulin resistance in mice (Wu et al., 2019).

It is noteworthy that Lactobacillus not only exerts a unique benefit in regulating gut flora but is also often found alongside *Akkermansia* in TCM interventions for T2DM. Fufang Fanshiliu Decoction (FFSLD) significantly increased the number of *Lactobacillus* and *Akkermansia* and decreased the number of potentially pathogenic bacteria (*Alistipes*, *Desulfovibrio*, and *Helicobacter*), as well as decreased serum and intestinal mucosal levels of pro-inflammatory levels of cytokines IL-1β, IL-6, TNF-α, and TGF-β, and regulated disorders of glucose and lipid metabolism, significantly improving symptoms of T2DM (Li L. et al., 2022). Ge-Gen-Jiao-Tai-Wan (GGJTW) increased the relative abundance of the beneficial bacteria *Lactobacillus* and *Akkermansia*, while decreasing the abundance of *Klebsiella* and *Bacteroides*, and significantly increased the levels of BAs, taurocholic acid (TCA), and glycoconjugate (GCA) levels. These bile acids promoted glucagon-like peptide-1 (GLP-1) secretion through activation of the activated bile acid-FXR/TGR5-GLP-1 pathway, which mediated significant improvement in T2DM symptoms (Chen et al., 2021). Shouhui Tongbian Formula (SHTB) modulates the metabolism of SCFAs and branched-chain amino acids (BCAAs) and up-regulates the IRS-1/PI3K/AKT signaling pathway by remodeling the structure of the intestinal microbial community, increasing the abundance of the beneficial bacteria *Akkermansia* and *Parabacteroides* and decreasing the abundance of the harmful bacteria, which leads to a reduction in the inflammatory response and improvement in insulin resistance (Wang T. et al., 2024). An experimental study by Chen et al. demonstrated that Simiao Wan (SMW) modulates BAs levels by remodeling gut microbial community structure, increasing the abundance of A*llobaculum, Akkermansia,* and *Lactobacillus*, decreasing the abundance of *Coprococcus* and *Halomonas*, and decreasing the 12α-OH/non-12α-OH BAs ratio, activate FXR and inhibit SREBP-1, thereby improving insulin resistance and hepatic lipid accumulation in T2DM mice (Han et al., 2021). Gao et al. found that Shenqi formula (SQ) reduced the abundance of the harmful bacteria *Ruminococcus* and *Phascolarctobacterium* by increasing the abundance of *Lachnospiraceae_NK4A136_group* and *Akkermansia* and corrected the testosterone-enoic acid and glycerophospholipid metabolic disorders to regulate lipid levels and reduce inflammatory responses (Gao et al., 2024).

These studies demonstrated that both GTG and DBD herbal combinations increased the relative abundance of *Lactobacillus* and *Akkermansia* and effectively improved T2DM. *Lactobacillus*, a gram-positive bacillus, and *Akkermansia*, a gram-negative anaerobic bacterium belonging to the phylum Verrucomicrobiales, are both promising applications for next-generation probiotic and postbiotic precursor strains (Vallianou et al., 2021; Jang et al., 2019). It has been demonstrated that these beneficial bacteria can enhance human immune function, promote digestion and absorption, reduce harmful bacteria, improve glycemic control, decrease insulin resistance, and reduce inflammatory response (Everard et al., 2013; Oh et al., 2020). Therefore, *Lactobacillus* and *Akkermansia* show good potential in improving T2DM and deserve further research and development.

#### 5.3.2 Improving metabolite regulation in T2DM

In T2DM, gut microbiota metabolites such as BAs, SCFAs, trimethylamine N-oxide (TMAO), and tryptophan metabolites play a crucial role (Tanase et al., 2020; Wu J. et al., 2023; Yan et al., 2022). These metabolites regulate host metabolic pathways and signaling, influencing insulin sensitivity, blood glucose control, and inflammatory responses (Yuan et al., 2021; Zhang Y. et al., 2023). For example, bile acids regulate glucose metabolism and energy homeostasis, influence insulin sensitivity, and the intestinal flora further influence lipid metabolism by altering bile acid composition via bile salt hydrolases (Yoo et al., 2022). Tryptophan is converted to indole derivatives by intestinal microbial action, which can affect the central nervous system and immune responses (Canfora et al., 2019).

PuRenDan (PRD) increases the abundance of beneficial bacteria such as *Prevotella* sp. *10 (H), Parabacteroides* sp. *SN4,* and *Flavobacteriales bacterium,* while decreasing the abundance of T2DM-associated harmful bacteria such as *Rickettsiaceae bacterium 4572_127, Psychrobacter pasteurii* and *Parabacteroides* sp. *CAG409*. Besides, PRD modulates the levels of key metabolites (pantothenic acid, 1-methylhistamine, and 1-methylhistidine) and their associated metabolic pathways, improving lipid metabolism, insulin resistance, and inflammatory responses, thus exerting its therapeutic effects on T2DM (Ma et al., 2024). Ma et al. found that Yu-Ye Tang (YYT) increased the abundance of *Lactobacillus, Candidatus_Saccharimonas, UCG-005, Bacteroides* and *Blautia* in the gut. It also reduced the abundance of the harmful bacteria *Allobaculum* and *Desulfovibrio*, while YYT exerted anti-inflammatory, metabolic modulating and ameliorative effects in T2DM rats by increasing the levels of tryptophan metabolites (L-tryptophan, L-kynurenine and 5-hydroxytryptophan) (Ma et al., 2023). Furthermore, it has been shown that Shenlian decoction (SL) increased *Bacteroides_acidifaciens* and decreased harmful bacteria (*Prevotellaceae, Rikenellaceae,* and *Helicobacteraceae*), as well as affected key metabolic pathways such as starch metabolism, pentose and glucuronide interactions and riboflavin metabolism, thereby reducing fasting blood glucose levels, protecting pancreatic β-cell function and improving metabolic function in T2DM mice (Sun et al., 2022).

Li et al. explored the ameliorative effects of a herbal formula (LLKL) containing *Edgeworthia gardneri, Sibiraea angustata,* and *Crocus sativus L* in T2DM rats. LLKL was able to significantly reduce the Firmicutes/Bacteroidetes ratio, decrease serum lipid polysaccharide (LPS) levels, and inhibit the Toll-like receptor (TLR) signaling pathway, including lowering the expression of TLR4, MyD88, and CTSK, to reduce the inflammatory response, thereby effectively improving insulin resistance and lowering blood glucose levels (Li M. et al., 2020). Huangqi Guizhi Wuwu Decoction (HGWD) ameliorates symptoms associated with T2DM, especially diabetic peripheral neuropathy (DPN), by modulating the gut microbiota, metabolites and key factors. HGWD significantly improves the diversity of intestinal microorganisms and lipid metabolites, thereby restoring the intestinal microbiological balance, and reduces oxidative stress by increasing the levels of antioxidants SOD and GSH, reducing MDA levels to alleviate oxidative stress, lowering the pro-inflammatory cytokines TNF-α, IL-1β, and IL-6 and increasing the levels of IL-10, NGF, and NT-3 to promote nerve repair and regeneration (Zhang K. et al., 2024). A study showed Gegen Qinlian Decoction (GQD) collectively ameliorated metabolic disorders in T2DM rats by increasing the abundance of *Faecalibacterium* and *Roseburia*, decreasing the abundance of deleterious bacteria, increasing the level of butyric acid in the feces, and decreasing the expression of immune-related genes in pancreatic islets, Nfkb1, Stat1, and Ifngr1, and insulin resistance (Xu et al., 2020). Another study on GQD showed that it increased the number of *Flavonifractor* and *Acetatifactor*, decreased the number of *Anaerofustis* and *Gammaproteobacteria*, and protected islet function in T2DM rats by decreasing the levels of CRP, IL-1β, TNF-α, and MCP-1, and by increasing the expression of tight junction protein ZO-1, occludin and claudin-1 expression (Tian et al., 2021).

**Figure 4:**
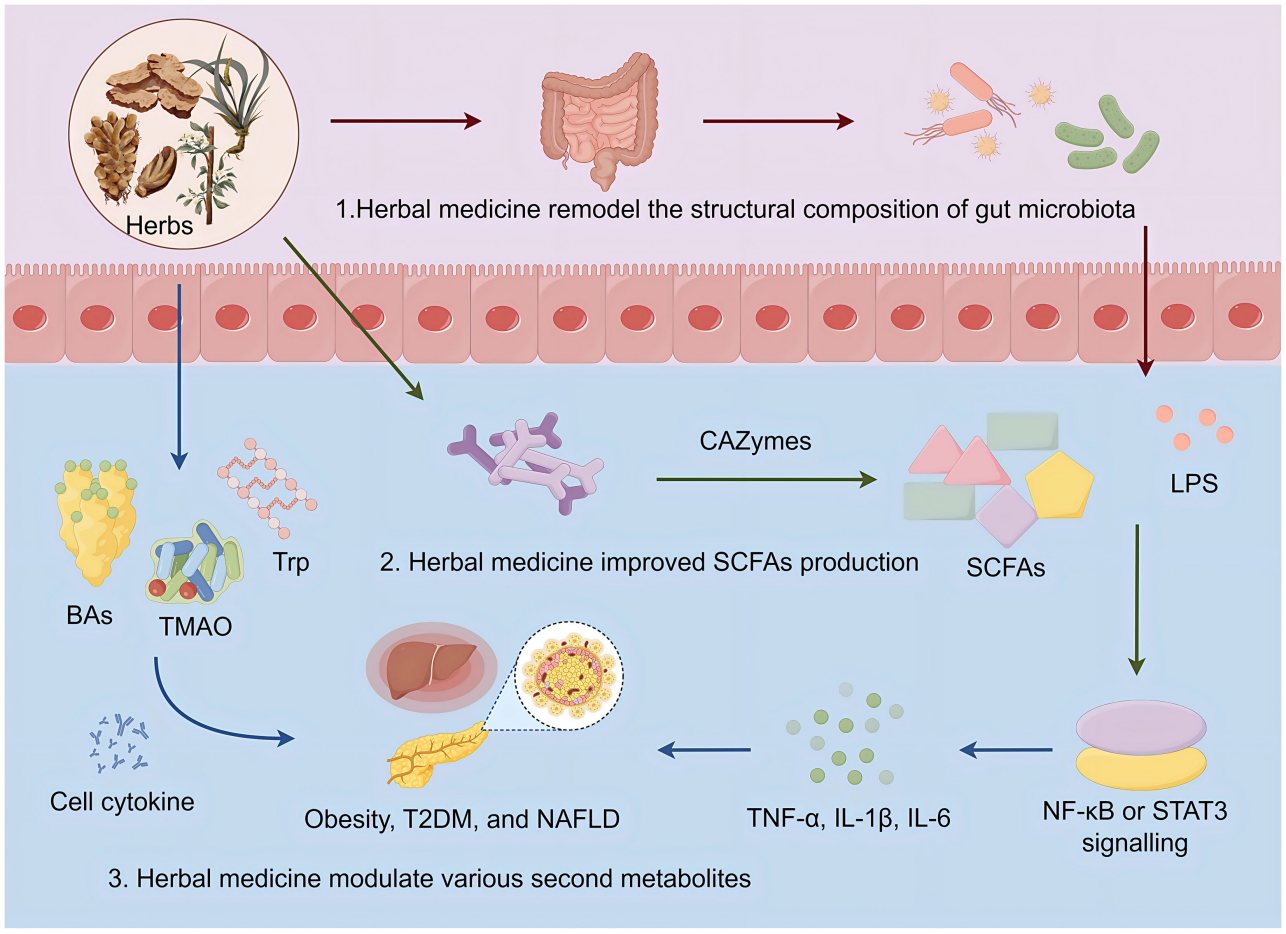
Multi-mechanistic pathways of herbal medicines in ameliorating metabolic diseases through gut microbiota modulation. (1) Remodeling gut microbiota structure: Herbal medicines alter the composition of intestinal microorganisms, reducing harmful bacteria and promoting beneficial bacteria, thereby restoring microbial ecological balance. (2) Enhancing SCFAs production: Herbal components serve as substrates for carbohydrate-active enzymes (CAZymes) produced by beneficial gut bacteria, leading to increased fermentation and SCFAs generation. These SCFAs strengthen intestinal barrier integrity and suppress inflammation. (3) Modulating microbial secondary metabolites: Herbal medicines regulate multiple bioactive metabolites including BAs, trimethylamine N-oxide (TMAO), and tryptophan (Trp) derivatives. BAs activate farnesoid X receptor (FXR) and Takeda G protein-coupled receptor 5 (TGR5) signaling to improve lipid metabolism and glucose homeostasis. Reduced LPS levels attenuate NF-κB or STAT3 signaling pathway activation, thereby decreasing pro-inflammatory cytokines (TNF-α, IL-1β, IL-6). The collective effects of these mechanisms ultimately improve obesity, T2DM, and NAFLD through enhanced immune cell cytokine regulation and restored metabolic homeostasis. This figure encapsulates the three pillars of herbal medicine action: structural remodeling (restoring ecological balance), functional enhancement (SCFA production), and metabolite modulation (BAs, TMAO, Trp derivatives). The integration of these pillars through immune cell cytokine regulation underscores that metabolic improvement is not merely a local gut effect but requires systemic immune-metabolic reprogramming by figdraw.com.

To provide a comprehensive visual summary of the mechanisms discussed above, Figure 4 illustrates the multi-level interplay between herbal medicines, gut microbiota, and metabolic homeostasis. This mechanistic framework integrates microbial structural remodeling, SCFAs production, and secondary metabolite modulation into a coordinated network that ultimately ameliorates metabolic diseases and its associated complications. While these multi-target mechanisms offer clear therapeutic advantages, it is important to place herbal medicine in context alongside conventional treatments. Orthodox medicines such as metformin, statins, and GLP-1 agonists provide precise single-target interventions with rapid onset and rigorous standardization, but they often fail to fully address the complex, interconnected nature of metabolic diseases and may cause adverse effects with long-term use (Lai et al., 2024; Abebaw et al., 2025). Herbal medicines, by contrast, take a more holistic approach: they modulate multiple body systems simultaneously through gut microbiota, offer favorable safety profiles for long-term use, and are more affordable (Ao et al., 2026; Pfuhlmann et al., 2025). However, they also face critical limitations—compositional variability due to geographic origin, harvest conditions, and processing methods; incomplete mechanistic understanding; insufficient large-scale clinical validation; and potential safety concerns including hepatotoxicity, nephrotoxicity, and clinically significant herb–drug interactions (Li et al., 2025).

### 5.4 Limitations in translating animal microbiome findings to humans and clinical translation considerations

A critical caveat in interpreting this review is the translational gap between animal models and human clinical application. Several factors limit the direct extrapolation of findings from rodent studies to human metabolic diseases: (1) Inter-species microbiota differences: The composition and function of the gut microbiota differ substantially between rodents and humans. For example, *Akkermansia muciniphila*, a bacterium frequently reported as increased by herbal interventions in mice, colonizes different mucosal niches in humans, and its metabolic responses may vary. The Firmicutes/Bacteroidetes ratio, often cited as an obesity marker in rodent studies, shows inconsistent associations with human metabolic disease. (2) Dietary and environmental confounders: Laboratory animals consume standardized, sterile diets and live in controlled environments, whereas human microbiota is shaped by diverse dietary patterns, geographic location, medication use, and lifestyle factors that are difficult to replicate or control. (3) Dosing and formulation challenges: Herbal doses used in animal studies (often 1–10 g/kg body weight) are frequently not scalable to human equivalents due to differences in metabolism, bioavailability, and tolerability. Standardization of herbal extracts remains a major barrier to clinical translation. (4) Immune system divergence: Rodent immune responses, particularly TLR4 signaling and inflammatory cytokine profiles, differ qualitatively and quantitatively from humans, potentially affecting the relevance of LPS/TLR4-mediated mechanisms observed in animal studies. (5) Lack of human validation: To date, few randomized controlled trials have directly tested whether herbal-induced microbiota changes in animals are reproducible in human subjects with metabolic diseases. The few available human studies show promising but inconsistent results, highlighting the need for well-powered clinical trials with microbiome endpoints. These translational limitations underscore that the mechanisms identified in this review should be considered hypothesis-generating rather than evidence of clinical efficacy. Future research must prioritize human intervention studies with standardized herbal preparations, multi-omics microbiota profiling, and clinically relevant metabolic endpoints.

While herbal medicines are generally perceived as safe, several safety concerns warrant attention. The immunomodulatory effects of herbal polysaccharides raise theoretical concerns in patients with autoimmune conditions or those receiving immunosuppressive therapy Rather than choosing one approach over the other, the best strategy may be to combine them. Conventional drugs can provide rapid control of metabolic symptoms, while herbal preparations can address the underlying gut dysbiosis and restore long-term metabolic balance. Therefore, future research should focus on establishing standardized protocols for herbal processing and quality control, identifying key microorganisms and active components using multi-omics techniques, and screening responsive patient populations through clinical trials. These efforts will support the development of standardized probiotics and postbiotics. Meanwhile, artificial intelligence and systems pharmacology will enable personalized therapeutic predictions, facilitating the integration of validated herbal therapies into routine clinical practice.

## 6 Conclusion

Metabolic diseases are complex pathological conditions caused by multiple factors, with insulin resistance as the central mechanism, and there is an urgent need to find effective treatments. Patients with obesity, NAFLD, and T2DM experience significant changes in the composition and function of their gut microbiota, including a decrease in beneficial bacteria, an increase in harmful bacteria, and alterations in metabolites such as SCFAs. In this review, we explore the role of herbal polysaccharides, non-polysaccharide components, and herbal formulations in improving metabolic diseases through the modulation of the gut microbiota. The herbs modulate the production of flora metabolites, especially *Lactobacillus* and *Akkermansia*, and promotes the production of beneficial metabolites such as SCFAs and BSCFAs, which improve insulin sensitivity and glycemic control by modulating the mTORC1-mediated IRS1/PI3K/AKT signaling pathway. Meanwhile, the herbal compound further improved insulin sensitivity and overall metabolic status by reducing the production of inflammatory mediators (IL-1, TNF-α, MyD88, and TLR-4) and attenuating the inflammatory response in the liver and other tissues. In addition, the herbal components influence intestinal absorption and transport functions and regulate intestinal barrier function and hepatic metabolism through activation of P-gp and modulation of FXR and other characteristic pathways. The multi-target and multi-pathway regulatory mechanism of PRD, SHTB, and LLKL herbal formulations makes it a potential natural drug. These compound formulas not only restored the intestinal microecological balance, but also improved insulin resistance, lipid metabolism disorders and inflammatory responses through the regulation of metabolites and signaling pathways, providing a new scientific basis for the treatment of T2DM. In summary, the regulation of the intestinal microenvironment by herb medicine is characterized by multi-target and multi-level synergistic actions. The multi-mechanistic features of TCM in modulating the gut microbiota for the treatment of T2DM demonstrate significant research value, providing more ideas and methods for the subsequent development of corresponding drugs.

## Supplementary material

SUPPLEMENTARY FIGURE S1 PRISMA 2020 flow diagram showing the systematic search and study.
