# Supplementary figures and images for: Herbal medicines modulate gut microbiota in metabolic diseases: a review
Source: Front Microbiol. 2026 Jun 25;17:1833443. doi: 10.3389/fmicb.2026.1833443 (PMC13346080; doi:10.3389/fmicb.2026.1833443)

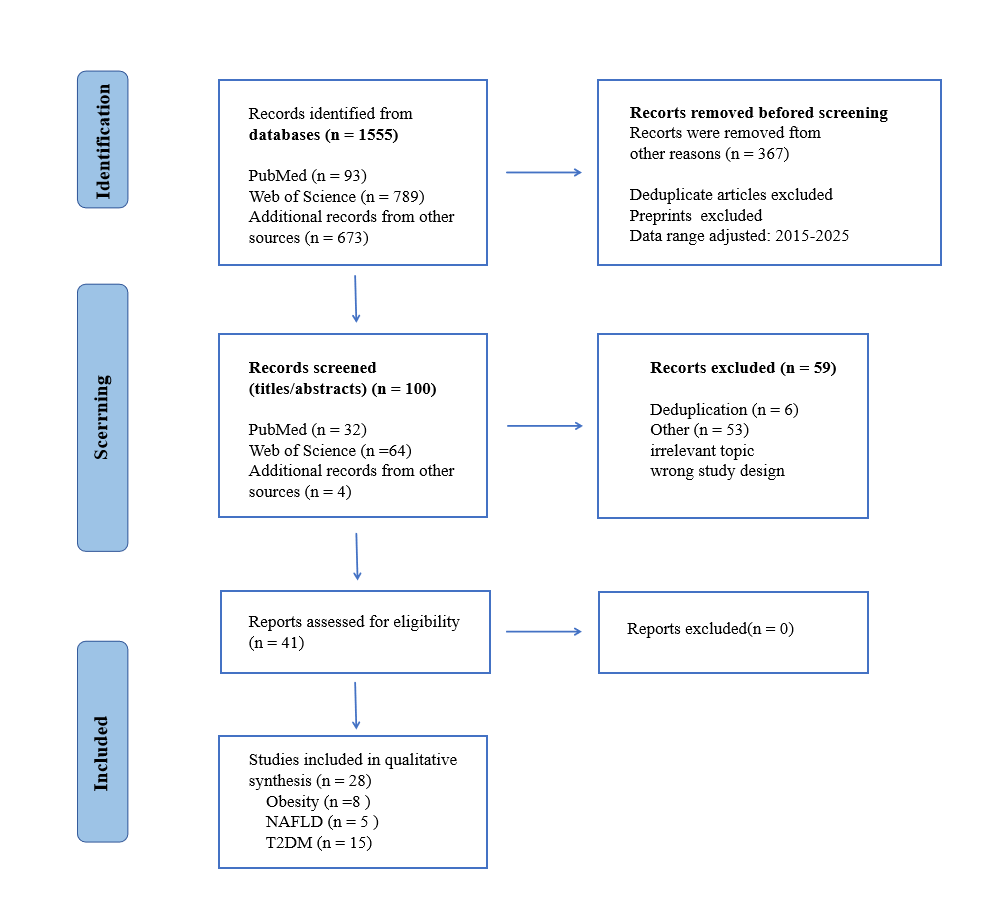

Supplement: SUPPLEMENTARY FIGURE S1 — PRISMA 2020 flow diagram showing the systematic search and study. [file Image_1.jpeg]
